# Reduced Medial Prefrontal Control of Palatable Food Consumption Is Associated With Binge Eating Proneness in Female Rats

**DOI:** 10.3389/fnbeh.2019.00252

**Published:** 2019-10-31

**Authors:** Elaine B. Sinclair, Kelly L. Klump, Cheryl L. Sisk

**Affiliations:** ^1^Neuroscience Program, Michigan State University, East Lansing, MI, United States; ^2^Department of Psychology, Michigan State University, East Lansing, MI, United States

**Keywords:** binge eating, medial prefrontal cortex, eating disorders, excitatory/inhibitory, palatable food intake, female rats

## Abstract

Binge eating is the core, maladaptive eating behavior that cuts across several major types of eating disorders. Binge eating is associated with a significant loss of control over palatable food (PF) intake, and deficits in behavioral control mechanisms, subserved by the prefrontal cortex (PFC), may underlie binge eating. Few studies, to date, have examined whether the PFC is directly involved in the expression of binge eating. As such, the present study investigated the functional role of the medial PFC (mPFC) in PF consumption, using an individual differences rat model of binge eating proneness. Here, we tested the hypothesis that binge eating proneness (i.e., high levels of PF consumption) is associated with reduced mPFC-mediated behavioral control over PF intake. In experiment 1, we quantified PF-induced Fos expression in both excitatory and inhibitory neurons within the mPFC in binge eating prone (BEP) and binge eating resistant (BER) female rats. In experiment 2, we pharmacologically inactivated the mPFC of BEP and BER female rats, just prior to PF exposure, and subsequently quantified PF intake and scores of feeding behavior. While most Fos-expressing neurons of the mPFC in both BEPs and BERs were of the excitatory phenotype, fewer excitatory neurons were engaged by PF in BEPs than in BERs. Moreover, pharmacological inactivation of the mPFC led to a significant increase in PF intake in both BEPs and BERs, but the rise in PF consumption was stronger in BEPs than in BERs. Thus, these data suggest that lower, PF-induced excitatory tone in the mPFC of BEP rats may lead to a weaker, mPFC-mediated behavioral “brake” over excessive PF intake.

## Introduction

Binge eating, defined as the consumption of a large amount of food (typically palatable food, PF), in a short period of time, is the core, maladaptive eating behavior that cuts across nearly every major type of eating disorder (ED) that predominantly affects women (Hudson et al., 2010). Binge eating is associated with a loss of control over food intake (American Psychiatric Association, 2013) and women with binge eating-related EDs [i.e., bulimia nervosa (BN), binge eating disorder (BED), sub-threshold EDs] possess several trait characteristics that suggest deficits in behavioral control mechanisms, including high impulsivity, behavioral rigidity, and co-morbid substance abuse and dependence (Calero-Elvira et al., 2009; Juarascio et al., 2015; Lee-Winn et al., 2016). Because the prefrontal cortex (PFC) subserves the core executive functions that are disrupted in women with binge eating-related EDs, dysfunctional, PFC-mediated behavioral regulation may be an etiologic factor in the development of such disorders.

Neuroimaging studies in women provide compelling support for the notion that disturbances in behavioral regulation are associated with BN, BED, and general binge eating. Specifically, women with BN and BED consistently demonstrate diminished PFC activation during tasks where behavioral regulation and behavioral inhibition are required (Wu et al., 2013). For example, women with BN and BED display reduced activation of select PFC sub-regions during the Simon Spatial Incompatibility task (assessing self-regulatory control), and the magnitude of PFC activation during task performance in women with BN negatively correlates with scores of BN pathology (Marsh et al., 2009, 2011). In addition, ventromedial PFC activation is lower in women with BED during the Stroop color-word interference task as compared to their non-BED counterparts (Balodis et al., 2013), and self-reported levels of dietary restraint (e.g., reported efforts to control food intake) negatively correlate with the magnitude of PFC activation during task performance in the BED group. Combined, these data suggest that the striking abnormalities in eating behavior inherent to binge eating may be driven by deficient, PFC-mediated control over food intake.

Though reduced PFC neural activity seems to underlie the executive control deficits (i.e., high impulsivity, behavioral rigidity) seen in women with EDs, imaging studies in humans have yet to identify whether dysfunctional PFC activity plays a causal role in the expression of eating pathology (i.e., binge eating). Animal models are invaluable tools for gaining insight into the mechanistic role for PFC circuitry in binge eating. To date, several lines of research in animal models have investigated the functional significance of the medial PFC (mPFC) to PF consumption. In general, the mPFC appears to exert regulatory control over PF intake: pharmacological inactivation of the ventral mPFC leads to a significant increase in the amount of time rats spend engaged in bouts of PF consumption (Mena et al., 2011; Baldo et al., 2015), and pharmacological inactivation of the prelimbic sub-region of the mPFC significantly increases high fat diet intake (Corwin et al., 2016). Thus, at baseline, the mPFC appears to serve as a behavioral “brake” over excessive PF intake. Further, the large population of excitatory projection neurons of the mPFC appears to subserve this mPFC-mediated behavioral brake, as 80–90% of all mPFC neurons that are engaged (i.e., express the neural activation marker c-fos) by PF intake are of the excitatory neuron phenotype (Gaykema et al., 2014). Thus, in general, PF intake in rodents appears to be tonically inhibited by excitatory projection neurons within the mPFC. However, no studies to date have investigated whether variations in the ability for mPFC excitatory neurons to regulate PF intake are associated with eating pathology, *per se*. That is, one question that remains is whether a greater propensity to engage in high amounts of PF consumption (i.e., binge eat) stems from a weaker “hold” over PF intake by excitatory neurons within the mPFC.

To gain initial insight into the functional relevance of the mPFC for eating pathology, our lab has previously identified patterns of PF-induced, neural activation (i.e., Fos expression) within the mPFC using an individual differences rat model of binge eating proneness. In this model, binge eating prone (BEP) and binge eating resistant (BER) rats are identified based on consistently high vs. consistently low intake of intermittently presented PF, respectively. Notably, the behavioral differences between BEPs and BERs mirror key differences between binge eaters and non-binge eaters in the human condition: BEPs consume significantly more PF than do BERs, BEPs, and BERs do not differ in either chow intake or body weight, and, behaviorally, BEPs display a greater loss of control over PF intake than do BERs (Klump et al., 2011a, b, 2013; Oswald et al., 2011; Hildebrandt et al., 2014; Sinclair et al., 2015). Using this model, our lab quantified PF-induced Fos expression within the cingulate, prelimbic, and infralimbic sub-regions of the mPFC in BEP and BER female rats, and found significantly higher Fos expression within each sub-region of the mPFC in BEPs as compared to BERs (Sinclair et al., 2015). Thus, our data suggested that differential engagement of the mPFC, in the presence of PF, correlates with binge eating proneness in female rats (Sinclair et al., 2015).

The aim of the present study was to further delineate the functional relevance of the mPFC to binge eating proneness in this model. Given that excitatory neurons of the mPFC appear to serve as a behavioral brake over PF intake, we hypothesized that (1) fewer excitatory neurons would be engaged by PF (i.e., express Fos) in BEP vs. BER female rats, and (2) the mPFC-mediated behavioral brake over PF consumption would be weaker in BEP vs. BER female rats. To test this hypothesis, we first quantified Fos expression in mPFC excitatory neurons of BEPs and BERs using double-label immunohistochemistry in Experiment 1. In Experiment 2, we used the GABA-A agonist muscimol to pharmacologically inactivate the mPFC of BEPs and BERs and then quantify the associated changes in PF intake, chow intake, and scores of feeding behavior. Our hypothesis predicted that BEPs would have lower PF-induced Fos expression in mPFC excitatory neurons as compared to BERs, and that pharmacological inactivation of the mPFC would yield a larger increase in PF intake in BEPs than in BERs. Such results would provide preliminary evidence that binge eating proneness may be at least partially driven by weaker mPFC-mediated control over hedonic, reward-driven feeding.

## Materials and Methods

### Experiment 1, PF-Induced Fos Expression in Excitatory and Inhibitory Neurons of the mPFC in BEP and BER Female Rats

#### Animals and Housing

A total of 100 young adult (postnatal day 60) female, Sprague-Dawley rats were obtained from Harlan Laboratories (Madison, WI, United States) and were run in two separate cohorts of *N* = 70 rats (cohort 1) and *N* = 30 rats (cohort 2). Upon arrival, rats were individually housed in clear Plexiglass cages (45 cm × 23 cm × 21 cm) with enrichment and *ad libitum* access to chow (Harlan Teklad Global Diets: 8640, Madison, WI, United States) and water. Rats were maintained on a 12:12 reverse light-dark cycle with lights out at 10:00 AM, and were treated in accordance with the NIH Guide for the Care and Use of Laboratory Animals. All animal procedures were approved by the Michigan State University Institutional Animal Care and Use Committee.

#### Feeding Tests

For both cohorts, feeding tests for experiment 1 began after 1 week of acclimation to housing conditions at our facility, so all testing in each cohort began on postnatal day 67. Feeding tests were run in two separate cohorts of rats and were conducted using a protocol adapted from one that has been used previously in our lab (Klump et al., 2011a, b, 2013; Hildebrandt et al., 2014; Sinclair et al., 2015; Culbert et al., 2018). Feeding tests were conducted over a period of 2 weeks and included six total feeding test days. Feeding test days occurred on MWF and consisted of 4 h of access to PF (∼25 g of Betty Crocker^®^ creamy vanilla frosting; 4.24 kcal/gm). PF was provided ∼10 min prior to lights out via hanging food dishes in the home cages; standard rat chow (50–70 g on cage tops) remained freely available during the PF exposure period. PF and chow were weighed at the beginning of the feeding test and again after 4 h of access using a standard electronic balance. Any remaining PF at the end of 4 h was removed from home cages until the next feeding test day, but chow remained freely available. On both feeding test days and non-feeding test days (i.e., days when PF was not provided), body weights and 24 h chow consumption were measured and recorded just before lights out.

#### BEP/BER Classification

Identification of BEP and BER rats followed protocols previously published by our lab (Klump et al., 2011a, b, 2013; Hildebrandt et al., 2014; Sinclair et al., 2015) using a tertile approach based on the 4 h PF intake values from each of the six feeding test days. The 4 h intake values were used for identification of binge eating phenotypes, given that binge eating can be readily observed in animals within this discrete window of PF exposure (Boggiano et al., 2007; Klump et al., 2011a, b, 2013; Hildebrandt et al., 2014). Four-hour PF intake values from each feeding test day were divided into top, middle, and bottom tertiles; each rat scored within one of the three tertiles on each feeding test day. Rats were classified as BEP if they scored within the highest tertile on at least three of the six (≥50%) feeding test days and never in the lowest tertile; rats were classified as BER if they scored within the lowest tertile on at least three of the six feeding test days and never in the highest tertile^1^. Table 1 provides the sample sizes and the proportions of BEPs and BERs that were identified in cohorts 1 and 2 for experiment 1.

**TABLE 1:** Proportions of BEP and BER rats identified in experiments 1 and 2.

**Experiment 1**			

**Phenotype**	**Cohort 1 (*N* = 70)**	**Cohort 2 (*N* = 30)**	
BER	14/70(20%)	8/30(27%)	
BEP	21/70(30%)	8/30(27%)	

**Experiment 2**			

**Phenotype**	**Cohort 1 (*N* = 20)**	**Cohort 2 (*N* = 30)**	**Cohort 3 (*N* = 29)**

BER	3(15%)	7(23%)	6 (21%)
BEP	3(15%)	9(30%)	6 (21%)

*All rats for experiments 1 and 2 arrived in the lab on postnatal 60, and all testing for both experiments began on postnatal day 67, following 1 week of acclimation to housing conditions.*

#### Induction of Fos Expression in the mPFC

One to three days following the final feeding test, all BEPs and BERs from cohorts 1 and 2 were given an additional 1 h of access to PF (∼25 g vanilla frosting) in their home cages in order to induce Fos expression. The Fos induction paradigm began at lights out, in order to emulate feeding test conditions (i.e., exposure to PF at the onset of the dark cycle) as closely as possible. Chow and water remained in each rat’s cage for the duration of the 1 h period of access to PF. At the end of 1 h, remaining PF was removed from each rat’s cage, and 30 min later, all BEPs and BERs were given a lethal dose of sodium pentobarbital (Fatal Plus^®^, 150 mg/kg i.p.). Thereafter, BEPs and BERs were intracardially perfused with a buffered saline rinse for 15 min followed by 4% paraformaldehyde for 20 min. In addition, we randomly selected a group of 10 binge eating neutral rats (i.e., non-BEP/non-BER) from cohort 1 to serve as a “No PF” control group for Fos expression. The “No PF” control rats were simply removed from their home cage, without any PF exposure, 30 min following lights out and were given a lethal dose of sodium pentobarbital (150 mg/kg) before intracardial perfusion. Following intracardial perfusion in all rats, brains were harvested, post-fixed over-night in 4% paraformaldehyde, and stored in 20% sucrose until sectioning. Brains were then cryostat-sectioned at 40 μm into four series and tissue sections were stored at −20°C until further processing for single and double label Fos immunohistochemistry.

#### Single Label Immunohistochemistry for Fos Expression in PF-Exposed and No PF Control Rats

One series of tissue sections through the mPFC from the “No PF” control rats (*N* = 10) in cohort 1, and from the PF-exposed, BEP and BER rats (*N* = 25) in cohort 1 were processed for single-label Fos immunohistochemistry. Fos immunohistochemistry was performed according to protocols previously published in our lab (Sinclair et al., 2015, 2017). Reagents and incubation times used for immunohistochemical detection of single-label Fos expression are presented in Supplementary Tables S2A,B.

#### Double Label Fos Immunohistochemistry in Excitatory and Inhibitory Neurons of the mPFC

The aim of Experiment 1 was to quantify Fos expression in excitatory neurons of the mPFC, to test our primary hypothesis that fewer excitatory neurons would be engaged by PF in BEPs as compared to BERs. However, inhibitory neurons of the mPFC also play a significant role in several executive functions of the mPFC, and inhibitory neurons of the mPFC are strongly engaged by PF intake in male mice (Gaykema et al., 2014). Thus, Experiment 1 quantified PF-induced Fos expression in both excitatory and inhibitory neurons of the mPFC, using several double-label immunohistochemical protocols outlined below.

##### Immunohistochemical detection of Fos expression in mPFC excitatory neurons

One series of tissue sections from all BEPs (*N* = 21) and BERs (*N* = 14) from cohort 1 of Experiment 1 were processed for dual labeling of Fos and special AT-rich sequence binding protein 2 (Satb2), a nuclear marker for excitatory projection neurons of the mPFC (Huang et al., 2013; Gaykema et al., 2014), using a sequential, double-label immunofluorescence protocol. Immunofluorescence was used for this protocol, given that both Fos and Satb2 are nuclear proteins. Specifically, two anatomically matched tissue sections from the caudal half of the mPFC from each BEP and BER rat were processed for Fos-Satb2 immunofluorescence. All rinses were performed using Tris-Buffered Saline (TBS) and all antibody solutions were made in TBS containing 0.3% Triton X-100 with 2% normal goat serum and 5% bovine serum albumin. Reagents and incubation times used for Fos-Satb2 immunofluorescence are presented in Supplementary Tables S2A,B. After final rinses, stained sections were mounted onto glass slides, air-dried, and coverslipped with *SlowFade*^®^ Gold Antifade Mountant (ThermoFisher Scientific). Of note, tissue sections from one BER and two BEPs were omitted from quantification due to poor staining quality, yielding a final sample size of *N* = 13 BERs and *N* = 19 BEPs for the final Fos-Satb2 analyses.

##### Immunohistochemical detection of Fos expression in mPFC inhibitory neurons

Additional series of tissue sections through the mPFC of BEPs and BERs from cohort 1 of experiment 1 were processed for dual labeling with Fos and either parvalbumin (PV, *N* = 11 BEP, *N* = 7 BER) or somatostatin (SOM, *N* = 10 BEP, *N* = 7 BER). Further, tissue sections from all BEPs (*N* = 8) and all BERs (*N* = 7) of cohort 2 in experiment 1 were processed for dual labeling with Fos and vasoactive intestinal peptide (VIP). We chose PV, VIP, and SOM to label mPFC inhibitory neurons, as PV-immunopositive (PV^+^), VIP-immunopositive (VIP^+^), and SOM-immunopositive (SOM^+^) interneurons, combined, account for up to 85% of the total inhibitory neuron pool within the mPFC (Kawaguchi and Kubota, 1997; Kawaguchi and Kondo, 2002). Tissue sections were double-labeled for Fos-PV, Fos-VIP, or Fos-SOM using a sequential, double-labeling protocol for brightfield imaging. All rinses were performed using TBS and all antibody solutions were made in TBS containing 0.3% Triton X-100 and 2% normal goat serum. Reagents and incubation times for all double labeling protocols are presented in Supplementary Tables S2A,B. After final rinses for each double-labeling protocol, stained sections were mounted onto glass slides, air-dried, dehydrated using a series of ethanols, and coverslipped.

#### Quantification of Single and Double-Label Fos Expression in the mPFC

##### Single label Fos quantification

Quantification of single and double label Fos immunohistochemistry were performed in all three sub-regions of the mPFC, the cingulate (CG), prelimbic (PL), and infralimbic (IL) cortices. Quantification of the total number of single-label, Fos-immunoreactive (Fos-ir) cells in the mPFC followed protocols previously published in our lab (Sinclair et al., 2015). The total numbers of Fos-ir cells in each sub-region of the mPFC were estimated using unbiased stereological methods, with the optical fractionator probe in Stereoinvestigator (Microbrightfield Biosciences, Willingston, VT, United States), under brightfield illumination. Stereological parameters that were used in the present study are described in detail elsewhere (Sinclair et al., 2015).

##### Fos-Satb2 quantification

Quantification of Fos-Satb2 immunofluorescence was performed under epi-illumination using an Olympus BX51 microscope equipped with a mercury arc lamp and both FITC and TRITC filters. Two sections through the caudal half of the mPFC were analyzed for each animal, with sections corresponding to plates 11 and 12 (+3.00 to +2.76 mm from bregma) of the Paxinos and Watson rat brain atlas (Paxinos and Charles, 2005). CG, PL, and IL sub-regions of the mPFC were traced relative to the anterior forceps of corpus callosum (fmi) under a 4× (NA 0.13, UPlanFl) air objective, and counts were performed with a 40× (NA 0.85, UPlanApo) air objective using the meander scan function in Neurolucida (Version 7, Microbrightfield, Willingston, VT, United States). Single labeled Fos^+^ cells and double labeled Fos^+^/Satb2^+^ cells were counted in each mPFC sub-region, in order to identify (1) the proportion of all Fos^+^ neurons that co-localized with Satb2, and (2) the total number of Fos^+^/Satb2^+^ cells within the mPFC. For proportional analyses in each mPFC sub-region, the total numbers of Fos^+^/Satb2^+^ cells were first calculated by summing across both hemispheres and across each tissue section. The total number of Fos^+^/Satb2^+^ cells was then divided by the total number of Fos^+^ cells (across each tissue section and across both hemispheres) to obtain a proportion.

##### Fos-PV, Fos-VIP, and Fos-SOM quantification

Quantification of Fos-PV, Fos-VIP, and Fos-SOM double label immunohistochemistry was performed under brightfield illumination using an Olympus BX51 microscope and Neurolucida, following the protocol outlined above in section “Immunohistochemical Detection of Fos Expression in mPFC Excitatory Neurons.” Single labeled Fos^+^ cells and double labeled Fos^+^/PV^+^, Fos^+^/VIP^+^, or Fos^+^/SOM^+^ cells, were counted in each mPFC sub-region, in order to identify (1) the proportions of all Fos^+^ cells co-localized with each inhibitory neuron marker, and (2) the total numbers of inhibitory neurons that were activated by PF. The proportions of all Fos^+^ cells that were co-localized with PV, SOM, or VIP were calculated as outlined above for Satb2.

### Experiment 2, Changes in PF Intake Following Pharmacological Inactivation of the mPFC in BEP and BER Female Rats

#### Animals and Housing

A separate set of 79 young adult (postnatal day 60) female, Sprague-Dawley rats were obtained from Harlan Laboratories (Madison, WI, United States) and were run in three separate cohorts of *N* = 20 (cohort 1), *N* = 30 (cohort 2), and *N* = 29 (cohort 3) rats (see Table 1). All rats were housed as described in Experiment 1 (See section “Animals and Housing”). For experiment 2, lights out occurred at 11:00 AM, 12:00 PM, or 2:00 PM, depending on the cohort. Animals were treated in accordance with the NIH Guide for the Care and Use of Laboratory Animals, and all protocols were approved by the Michigan State University Institutional Animal Care and Use Committee.

#### Feeding Tests and BEP/BER Classification

As in experiment 1, feeding tests for experiment 2 began after 1 week of acclimation to housing conditions in our facility, so all testing for each cohort began on postnatal day 67. Feeding tests for experiment 2 were run in three separate cohorts of rats and were conducted using the same protocol outlined above (See section “Feeding Tests”), except PF and chow were measured after both 1 and 4 h of access. PF and chow were measured at the additional 1 h time point, because we were interested in analyzing this earlier time point of access during the intra-mPFC muscimol infusions later in the study. Specifically, we predicted that the strongest changes to PF and/or chow intake following intra-mPFC muscimol infusions would occur soon after the infusion, given that the effects of muscimol begin almost immediately (Allen et al., 2008). Therefore, we included this 1 h time point, here, to ensure that significant differences in PF intake between BEPs and BERs are apparent prior to 4 h of exposure. To identify BEPs and BERs, we used PF intake data from the 4 h time point (as outlined above in section “BEP/BER Classification”^2^), to remain consistent with past studies in the lab (Klump et al., 2011a, b, 2013; Sinclair et al., 2015).

#### Stereotaxic Surgical Procedures

Bilateral guide cannulae were stereotaxically implanted to target the ventral half of the mPFC in all BEPs (*N* = 16) and BERs (*N* = 18) of experiment 2, and surgical procedures were completed 1–2 days following the final feeding test day. Rats were anesthetized via inhaled isoflurane and were secured in a Kopf stereotaxic frame. After exposing the skull using aseptic techniques, bilateral stainless steel guide cannulas (2.5 mm long, 26 gauge, Plastics One, Roanoke, VA, United States) were inserted at a 19° angle from vertical into the mPFC (+2.6 mm AP, ± 2.0 mm ML, −2.5 mm DV, relative to the skull surface). Guide cannulas were lowered into the mPFC such that the top of the cannulas was flush with the skull and the cannula tip landed 2.5 mm above the targeted infusion site of −5.0 mm from the skull surface (i.e., the ventral half of the mPFC). Guide cannulas were anchored to the skull and stainless-steel dummy cannulas (2.5 mm long, Plastics One) were inserted into guide cannulas to prevent blockage. Rats were given 5 mg/kg s.c. ketoprofen for pain management, 5 mg/kg of s.c. enrofloxacin antibiotic, and 1 mL of s.c. sterile saline at the time of surgery. Following surgery, animals were returned to their home cages on a warm heating pad and were monitored until awakening. Thereafter, rats were allowed a minimum of 5 days of recovery before any additional testing. During recovery, daily body weights were recorded, and rats were handled daily.

#### Intra-mPFC Infusions of Muscimol

Muscimol was obtained from Sigma-Aldrich and was diluted with 0.9% sterile saline to make a 10 mg/mL stock solution. The 15 ng and 30 ng doses of muscimol that were used for intra-mPFC infusions were prepared from the stock solution prior to infusions.

Infusions began ∼60 min before lights out to ensure that all rats were infused in the light phase and before PF was delivered. Two days prior to the first infusion of muscimol or saline vehicle, all rats were given one sham infusion to acclimate rats to necessary handling procedures. Two days later, muscimol infusions began following a within-subjects design: each BEP and BER rat received all three doses of muscimol: 0 ng (sterile saline), 15 ng, and 30 ng. Infusions occurred on alternating days of the week. During each infusion, rats were gently held in the lap of the experimenter as infusion cannulas were lowered into guide cannulas, and 0.5 uL of drug was delivered at a rate of 0.25 uL/min using a 5 μL Hamilton syringe and an automated microsyringe pump. Cannulas were held in place for one additional minute to prevent backflow of drug, after which rats were returned to their home cage until the start of the feeding test. The order in which each drug dose was delivered was randomized across each animal and across each testing day, and experimenters were blind to the dose of the drug during all infusions. Upon the completion of all infusions on each testing day (i.e., ∼10 min before lights out), feeding tests were conducted following the paradigm outlined in section “Feeding Test and BEP/BER Classification”. For experiment 2, all of our analyses focused on the 1 h time point after each infusion, but PF and chow intake were also measured after 4 h of exposure, to remain consistent with the initial feeding test period outlined in section “Feeding Test and BEP/BER Classification”.

#### Analysis of Feeding, Locomotor, and Grooming Behavior

During each feeding test that followed intra-mPFC infusions, a random sample of BEPs (*N* = 14) and BERs (*N* = 14) were video recorded in order to score feeding, locomotor, and grooming behavior, during the first hour of access to PF. Our assessment of feeding behavior, here, allowed us to determine if certain structural components of PF intake were altered by pharmacological inactivation of the mPFC. Moreover, the analysis of locomotor and grooming behavior ensured that pharmacological inactivation of the mPFC did not unduly affect either motor function or typical behaviors (i.e., grooming) that would be displayed by a rat in their home-cage environment. Rats were video recorded for the first hour of access to PF and recordings were scored using an event recorder by an experimenter blind to both binge phenotype and drug dose. Feeding behavior was scored for the full duration of the 1 h test, while locomotor and grooming behaviors were scored for only the first 30 min of the 1 h access period. Rats displayed the highest frequency of both locomotor and grooming behaviors during this initial 30-min time period. The following behaviors were quantified for each feeding test:

(1) Feeding behavior: latency to begin consuming PF, number of episodes of PF consumption, mean duration of all episodes of PF consumption, and total time spent consuming PF (i.e., the sum of all feeding episodes across the hour).
(2) Locomotor behavior: total number (counts) of cage crossings (i.e., moving past the midline of the cage in either direction) or rears.
(3) Grooming behavior: total number of grooming maneuvers (counts), including bilateral head sweeps and full body sweeps.

#### Verification of Cannula Placement Within the mPFC

Two days after the final drug infusion, all BEPs and BERs were intracardially perfused as outlined in section “Induction of Fos Expression in the mPFC”. Harvested brain tissue was cryostat sectioned at 40 μm into four series and mounted onto glass slides. Thereafter, sections were processed for cresyl violet staining in order to verify cannula placement within the mPFC; cannula placement and cannula tracks are shown in Figure 4. Of note, data from two BER rats and one BEP rat were excluded from all statistical analyses in experiment 2, due to either unilateral or bilateral guide cannula occlusion or guide cannula misplacement. This resulted in a final sample size of *N* = 16 BER and *N* = 15 BEP rats.

### Statistical Analyses

SPSS Statistics software (version 24) was used for all statistical analyses in experiments 1 and 2, with the alpha level set to 0.05. For the initial feeding tests in both experiments 1 and 2, individual mixed design ANOVAs were used to compare PF intake, chow intake, and body weights between BEPs and BERs, with the within-subjects factor being test day and the between-subjects factor being binge eating phenotype (i.e., BER or BEP). Cohen’s *d* effect sizes were calculated for each BEP vs. BER comparison in order to provide a standardized measure of the magnitude of the mean differences between the two phenotypes. Cohen’s *d* effect sizes were interpreted as small (*d* = 0.20), medium (*d* = 0.50), or large (*d* = 0.80) (Jacob, 1988).

For single label Fos expression, total Fos-ir cell number was analyzed between our “No PF” control group and our PF-exposed group using separate, independent sample *T*-tests within each sub-region of the mPFC.

For double-label Fos immunohistochemistry in experiment 1, we analyzed (1) the proportions of total Fos^+^ cells expressing each neuronal marker in BEPs and BERs, and (2) the total numbers of double-labeled cells, for each neuronal marker, in BEPs and BERs. Proportions of Fos^+^ cells expressing each neuronal marker were compared between the two phenotypes using two-proportion *z*-tests, while the total numbers of double-labeled cells were compared between BEPs and BERs using individual analysis of covariance (ANCOVA) models in each brain region, with the total amount of PF consumed at sacrifice as the covariate. ANCOVA models were used to ensure that the amount of PF consumed prior to sacrifice did not unduly affect Fos expression. Finally, Cohen’s *d* effect sizes were calculated for each comparison listed above.

For the feeding tests following each intra-mPFC infusion in experiment 2, individual mixed design ANOVAs were used to analyze PF intake, chow intake, and each component of feeding, locomotor and grooming behavior; the between-subjects factor was binge eating phenotype while the within-subjects factor was drug dose. For all ANOVAs, significant main effects of drug dose were followed up by pairwise comparisons using a Bonferroni correction and collapsing across phenotype if no drug x phenotype interaction was present.

For measures of PF intake following each muscimol infusion in experiment 2, Cohen’s *d* effect sizes were also calculated within BEPs and BERs separately, in order to determine the magnitude of the change in PF intake following each muscimol infusion, as compared to saline, in each phenotype individually (Morris and DeShon, 2002). For measures of PF intake in experiment 2, we also calculated the percent change in PF intake, relative to saline, for each dose of muscimol.^3^ Thereafter, values of percent change in PF intake were compared between BEPs and BERs using individual ANCOVA analyses at each drug dose, with PF intake at saline as the covariate.

## Results

### Experiment 1, PF-Induced Fos Expression in Excitatory and Inhibitory Neurons of the mPFC in BEP and BER Rats

#### Differences in PF Intake, Chow Intake, and Body Weights Between BEP and BER Rats During the Initial Feeding Test Period

Results from the mixed design ANOVAs analyzing initial feeding test data for experiment 1 are shown in Table 2. As expected, PF intake was significantly higher in BEPs than in BERs in both experiments, but neither body weights nor chow intake differed between the two phenotypes in either experiment 1 or 2 (Klump et al., 2011a, b, 2013; Hildebrandt et al., 2014; Sinclair et al., 2015). Though 24 h chow consumption on non-feeding test days did not differ between BEPs and BERs, 24 h chow consumption on feeding test days was significantly higher in BERs vs. BEPs in both experiments 1 and 2, a finding consistent with previous work in our lab (Sinclair et al., 2015).

**TABLE 2:** Experiment 1, mean comparisons between BEP and BER rats on PF intake, chow intake, and body weights across the feeding test period.

		**BEP vs. BER mean**	
**Variable**	**Mean (SE)**	**comparisons**	
		***F*(1,49)**	**Cohen’s *d***
**Body weights (g)**			
BER	196.03 (1.61)	0.47	0.19
BEP	197.52 (1.43)		

**Feeding test days**			

**PF intake, 4 h (g)**			
BER	5.97 (0.14)	289.45^∗∗∗^	4.77
BEP	9.16 (0.12)		
**Chow intake, 4 h (g)**			
BER	2.46 (0.15)	0.35	0.16
BEP	2.58 (0.13)		
**Chow intake, 24 h (g)**			
BER	9.82 (0.25)	24.85^∗∗∗^	1.39
BEP	8.17 (0.22)		

**Non-feeding test days**			

**Chow intake, 24 h (g)**			
BER	13.67 (0.21)	3.52^†^	0.53
BEP	13.14 (0.19)		

*Cohen’s *d* interpretation: small, *d* = 0.20; medium, *d* = 0.50; large, *d* = 0.80; ^∗∗∗^*p* < 0.001; ^†^*p* < 0.10.*

#### Single Label Fos Expression in the mPFC

Palatable food exposure induced significantly greater Fos expression in all three sub-regions of the mPFC as compared to a non-PF (i.e., home cage exposure) stimulus (Supplementary Figure S1). *T*-tests for the comparison of Fos-ir cell number between the “No PF” and PF-exposed experimental groups were statistically significant in the CG (*t*(33) = −3.80, *p* = 0.001), the PL (*t*(33) = −5.43, *p* < 0.001), and in the IL (*t*(33) = −5.93, *p* < 0.001) cortices.

#### Proportions of Fos-Expressing Neurons Co-localized With Excitatory and Inhibitory Neuron Markers

**FIGURE 1:**
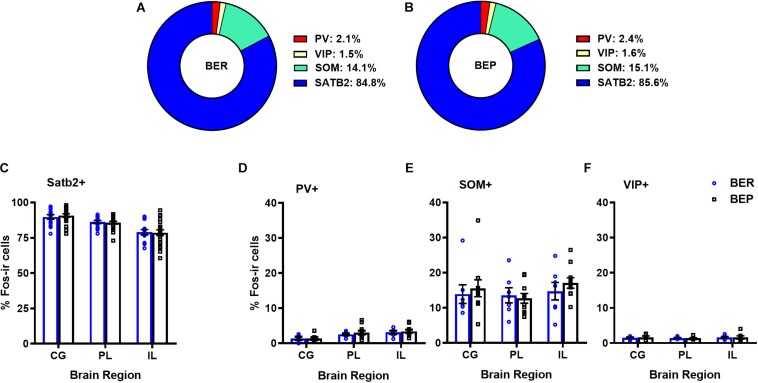
Proportions of mPFC excitatory and inhibitory neurons that express Fos during PF exposure in BEP and BER rats. Pie charts in **(A)** and **(B)** represent mean proportions (averaged across each mPFC sub-region) of all Fos+ neurons co-localized with excitatory and inhibitory neuron markers in BERs and BEPs. Bottom panel displays the proportions of Fos+ neurons, separated by mPFC sub-region, co-localized with excitatory neuron marker Satb2 **(C)** or inhibitory neuron markers PV **(D)**, SOM **(E)**, and VIP **(F)**. Error bars represent 1 SEM.

Overall, BEPs and BERs did not differ in the proportions of all Fos^+^ neurons that were of the excitatory phenotype (i.e., Satb2^+^) or of the inhibitory phenotype (i.e., PV^+^, VIP^+^, or SOM^+^) in any brain region (Figure 1 and Table 3). Regardless of binge eating phenotype, a majority of all Fos^+^ neurons were of the excitatory neuron phenotype (i.e., Satb2^+^, ∼85% in both BEPs and BERs), and much smaller proportions of Fos^+^ neurons were of the inhibitory neuron phenotype; on average, 2.2% were PV^+^, 14.6% were SOM^+^, and 1.6% were VIP^+^ across BEPs and BERs.

**TABLE 3:** Experiment 1, proportions of all Fos+ neurons co-localized with excitatory neuron marker Satb2 and inhibitory neuron markers PV, SOM, and VIP in BEPs and BERs.

		**BEP vs. BER mean**	
**Variable**	**Mean% (SE)**	**comparisons**	
		***z***	***p***

**Proportion Fos+ with Satb2**			
**Cingulate**			
BER	89.8 (0.02)	–0.17	0.86
BEP	91.5 (0.01)		
**Prelimbic**			
BER	85.8 (0.01)	–0.04	0.96
BEP	86.3 (0.01)		
**Infralimbic**			
BER	78.8 (0.02)	–0.03	0.97
BEP	79.2 (0.02)		

**Proportion Fos+ with PV**			

**Cingulate**			
BER	1.01 (0.41)	0.02	0.98
BEP	0.86 (0.33)		
**Prelimbic**			
BER	2.58 (0.29)	–0.06	0.95
BEP	3.07 (0.55)		
**Infralimbic**			
BER	2.66 (0.60)	–0.09	0.92
BEP	3.43 (0.45)		

**Proportion Fos+ with SOM**			

**Cingulate**			
BER	13.91 (2.65)	–0.09	0.92
BEP	15.56 (2.42)		
**Prelimbic**			
BER	13.56 (2.15)	0.05	0.96
BEP	12.69 (1.38)		
**Infralimbic**			
BER	14.73 (2.48)	–0.13	0.89
BEP	17.09 (1.50)		

**Proportion Fos+ with VIP**			

**Cingulate**			
BER	1.54 (0.20)	–0.01	0.98
BEP	1.66 (0.34)		
**Prelimbic**			
BER	1.44 (0.13)	0.01	0.99
BEP	1.38 (0.16)		
**Infralimbic**			
BER	1.66 (0.22)	0.01	0.99
BEP	1.59 (0.46)		

**z*-values represent two-proportion *z* tests.*

#### Total Numbers of Fos-Expressing Excitatory and Inhibitory Neurons in the mPFC of BEPs and BERs

**FIGURE 2:**
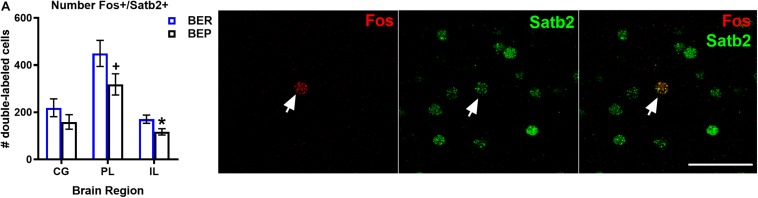
Total numbers of PF-activated excitatory neurons in the mPFC of BEPs and BERs. Values for # of double labeled cells in BEPs and BERs **(A)** represent ANCOVA-adjusted means, using PF consumed prior to sacrifice as the covariate, for each brain region. Error bars represent 1 SEM. Image panel depicts a Fos+ neuron co-localized with Satb2; scale bar represents 50 um. ^∗^*p* < 0.05, ^+^*p* < 0.09.

Although the proportions of Fos^+^ neurons that co-localized with Satb2 did not differ between BEPs or BERs in any sub-regions of the mPFC, ANCOVA analyses revealed that the total numbers of Fos^+^/Satb2^+^ neurons within the mPFC (co-varied by PF intake) was lower in the ventral mPFC (i.e., the PL and IL) of BEPs as compared to BERs (Figure 2 and Table 4). In the PL, the BEP vs. BER comparison of total Fos^+^/Satb2^+^ neuron number reached trend-level significance (*F*(1,29) = 3.10, *p* = 0.08) and was of medium effect size (*d* = 0.66). Furthermore, in the IL, total Fos^+^/Satb2^+^ neuron number was significantly lower in BEPs than in BERs (*F*(1,26) = 5.61, *p* = 0.02) with a large effect size (*d* = 0.92). Thus, the overall magnitude of excitatory neuron responsiveness to PF within the ventral mPFC was lower in BEPs as compared to BERs.

**TABLE 4:** Experiment 1, total numbers of Fos+ neurons co-localized with excitatory neuron marker Satb2 in the mPFC of BEPs and BERs.

		**BEP vs. BER mean**	
**Brain region**	**Mean # (SE)**	**comparisons**	
		***F* (df)**	**Cohen’s *d***
**Cingulate**			
BER	218.9 (37.93)	1.38 (1,29)	0.44
BEP	158.9 (30.82)		
**Prelimbic**			
BER	449.5 (55.47)	3.10^†^ (1,29)	0.66
BEP	318.1 (45.07)		
**Infralimbic**			
BER	170.8 (17.35)	5.61^∗^ (1,26)	0.92
BEP	117.1 (13.32)		

*Mean values represent ANCOVA-adjusted means using total PF consumed prior to sacrifice as the covariate. Effect sizes were calculated using ANCOVA-adjusted means; ^∗^*p* < 0.05; ^†^*p* < 0.09.*

**FIGURE 3:**
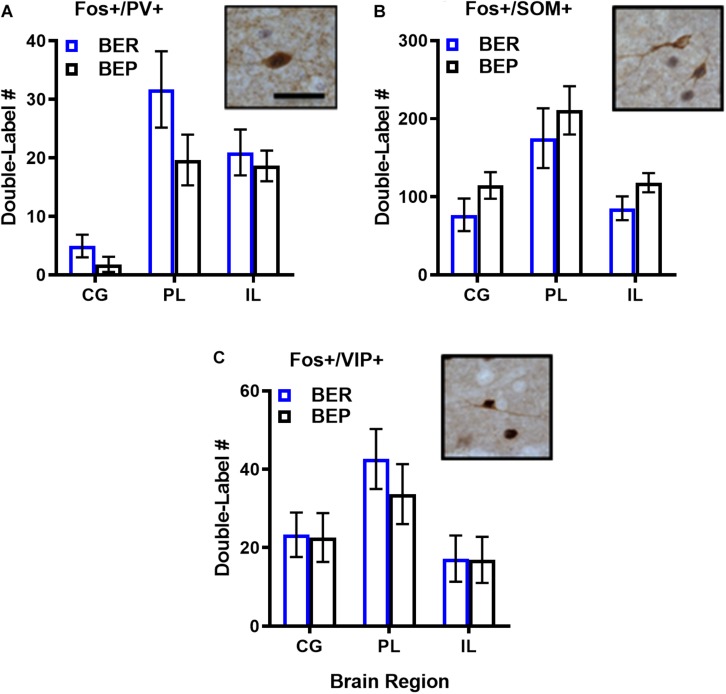
Total numbers of PF-activated inhibitory neurons in the mPFC of BEPs and BERs. Values for PV+ **(A)**, SOM+ **(B)**, and VIP+ **(C)** inhibitory neurons represent ANCOVA-adjusted means, using PF consumed prior to sacrifice as the covariate; error bars represent 1 SEM. Representative images of double-labeled inhibitory neurons are also presented in **(A–C)**; scale bar represents 50 pm.

As expected, the magnitude of inhibitory neuron responsiveness to PF did not differ significantly between BEPs and BERs in any brain region (*p*’s 0.18–0.66 for Fos-PV; *p*’s 0.65–0.85 for Fos-VIP; *p*’s 0.15–0.52 for Fos-SOM), but effect sizes for select BEP vs. BER comparisons were medium-to-large in magnitude (Figure 3 and Supplementary Table S4). First, the total number of Fos^+^/PV^+^ neurons was consistently higher in BERs than in BEPs, with large effect sizes in the CG (*d* = 1.77) and in the PL (*d* = 0.98). On the other hand, the total number of Fos^+^/SOM^+^ neurons was consistently higher in BEPs than in BERs, with a medium-to-large effect size in the CG (*d* = 0.68) and a large effect size in the IL (*d* = 0.88). Thus, despite a lack of significant differences between BEPs and BERs in overall inhibitory neuron responsiveness to PF, our data highlight cell-type specific patterns of PF-induced Fos expression in inhibitory neurons of the mPFC that differ between the two phenotypes.

### Experiment 2, Pharmacological Inactivation of the mPFC in BEP and BER Rats

#### Differences in PF Intake, Chow Intake, and Body Weights Between BEP and BER Rats During the Initial Feeding Test Period, Prior Intra-mPFC Muscimol Infusions

Results from the mixed design ANOVAs, analyzing feeding test data from the initial feeding test period in experiment 2 (i.e., where BEPs and BERs were initially identified), are presented in Table 5. As expected, PF intake was significantly higher in BEP vs. BER rats at both the 1 and 4 h time points, with no differences between BEPs and BERs in chow intake; body weights also did not differ between BEPs and BERs in experiment 2. As in experiment 1, 24 h chow consumption on non-feeding test days did not differ between BEPs and BERs, but 24 h chow consumption on feeding test days was significantly higher in BERs vs. BEPs.

**TABLE 5:** Experiment 2, mean comparisons between BEP and BER rats on PF intake, chow intake, and body weights across the initial feeding test period, prior to intra-mPFC drug infusions.

		**BEP vs. BER mean**	
**Variable**	**Mean (SE)**	**comparisons**	
		***F*(1,49)**	**Cohen’s *d***
**Body weights (g)**			
BER	200.85 (2.20)	3.09^†^	0.64
BEP	206.39 (2.27)		

**Feeding test days**			

**PF intake, 1 h (g)**			
BER	3.02 (0.22)	67.52^∗∗∗^	2.96
BEP	5.61 (0.23)		
**PF intake, 4 h (g)**			
BER	5.91 (0.26)	103.86^∗∗∗^	3.66
BEP	9.76 (0.27)		
**Chow intake, 1 h (g)**			
BER	1.17 (0.13)	0.00	0.01
BEP	1.17 (0.14)		
**Chow intake, 4 h (g)**			
BER	2.37 (0.19)	1.08	0.37
BEP	2.09 (0.20)		
**Chow intake, 24 h (g)**			
BER	9.73 (0.37)	9.25^∗∗^	1.10
BEP	8.10 (0.38)		

**Non-feeding test days**			

**Chow intake, 24 h (g)**			
BER	13.80 (0.38)	1.32	0.41
BEP	13.17 (0.39)		

*Mean values are based on data from BEPs (*N* = 15) and BERs (*N* = 16) that received intra-mPFC drug infusions in experiment 2; ^∗∗∗^*p* < 0.001; ^∗∗^*p* < 0.01; ^†^*p* < 0.10.*

#### Pharmacological Inactivation of the mPFC Increases PF Intake, but Not Chow Intake, in BEPs and BERs

**FIGURE 4:**
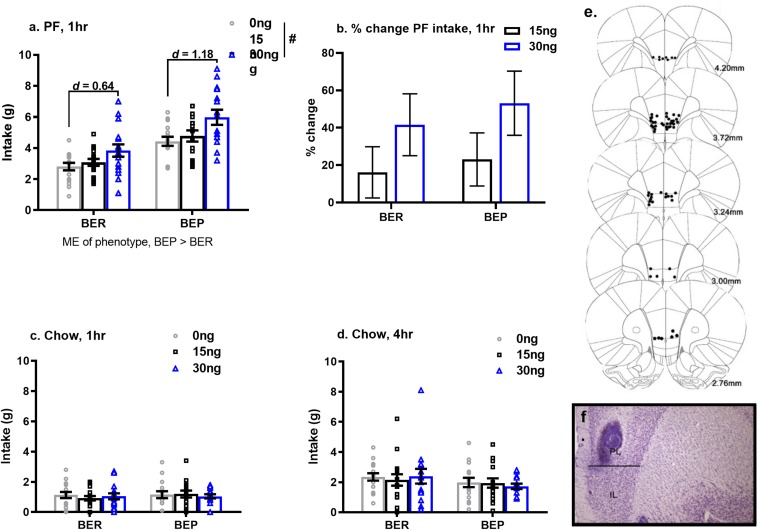
Pharmacological inactivation of mPFC enhances 1 h PF intake in BERs and in BEPs. Cohen’s *d* values in **(a)** represent the comparison of PF intake at saline to PF intake at 30 ng muscimol, calculated within each phenotype individually. Percent change in 1 h PF intake **(b)** was calculated relative to each rat’s individual PF intake value at saline. Group means in **(b)** represent ANCOVA-adjusted means, with each rat’s PF intake at saline as the covariate. Chow intake, after 1 h **(c)** and 4 h **(d)** of exposure, was unaffected by inactivation of the mPFC. Error bars in **(a–d)** represent 1 SEM. and doses of muscimol represent amount infused (in ng) into each hemisphere. Bilateral cannula placements along the rostro-caudal extent of the mPFC are shown in **(e)** and a representative image of cannula tracks within the mPFC is shown in **(f)**. ME, main effect; ^#^*p* < 0.05, main effect of drug.

As predicted, the effects of pharmacological inactivation of the mPFC on PF intake were strongest after 1 h (vs. 4 h) of PF exposure. Specifically, pharmacological inactivation of the mPFC induced a significant increase in 1 h PF intake in both BEPs and BERs (main effect of drug, *F*(2,58) = 13.13, *p* < 0.001, Figure 4a and Table 6), but no significant drug^∗^phenotype interaction (*F*(2,58) = 0.56, *p* = 0.57); between-phenotype differences in PF intake (BEP>BER) were maintained (main effect of phenotype, *F*(1,29) = 23.03, *p* < 0.001, Figure 4a). Follow-up, pairwise comparisons for the main effect of drug revealed that BEPs and BERs consumed significantly more PF following infusion of 30 ng muscimol as compared to both saline (*p* < 0.001) and 15 ng muscimol (*p* = 0.006). Specifically, 30 ng muscimol yielded a 41.57% increase in 1 h PF intake (compared to saline) in BERs and a 53.12% increase in BEPs (Figure 4b and Table 6). ANCOVA analyses, comparing the percent change in 1 h PF intake, revealed no significant differences between BEPs and BERs at either drug dose, but Cohen’s *d* effect size analyses, comparing 1 h PF intake after saline to 1 h PF intake after 30 ng muscimol, revealed a medium-to-large effect size in BERs (*d* = 0.64) but a large effect size in BEPs (*d* = 1.18, Table 6). Notably, the effect size for this comparison in BEPs was almost double that of BERs, suggesting that although inactivation of the mPFC enhanced 1 h PF intake in both phenotypes, the effect of mPFC inactivation on 1 h PF intake was stronger in BEPs than in BERs.

**TABLE 6:** Experiment 2, mean values and effect size estimates for 1 h PF intake and percent change in 1 h PF intake following pharmacological inactivation of the mPFC in BEP and BER rats.

				**Cohen’s *d***	
				**effect sizes**	
	**Mean (SE)**			**Saline vs.**	
**Variable**	**Saline**	**15 ng**	**30 ng**	**15 ng**	**30 ng**
**PF intake, 1 h (g)**					
BER	2.81 (0.24)	3.08 (0.23)	3.84 (0.40)	0.22	0.64
BEP	4.43 (0.29)	4.77 (0.36)	5.98 (0.49)	0.34	1.18

				**BEP vs. BER mean**	
				**comparisons**	
**Variable**	**Mean (SE)**			***F*(2,28)**	

**% change PF intake, 1 h**					

**15 ng muscimol**					
BER	16.13 (13.72)			0.11	
BEP	23.04 (14.21)				
**30 ng muscimol**					
BER	41.57 (16.57)			0.21	
BEP	53.12 (17.16)				

*Cohen’s *d* effect size calculations were calculated for each drug dose in each phenotype separately. Percent change in PF intake at each drug dose is relative to each rat’s individual PF intake at saline; mean values for % change in PF intake represent ANCOVA-adjusted means, with PF intake at saline as the covariate.*

At the 4 h time point, the observed changes in PF intake were weaker than those observed after 1 h, likely due to metabolism of muscimol by 4 h, but the pattern of results was similar to that seen at the 1 h time point. Chow intake was unaffected by pharmacological inactivation of the mPFC, and neither 1 h nor 4 h chow intake differed between BEPs and BERs (Figures 4c,d).

#### Pharmacological Inactivation of the mPFC Alters the Structure of Feeding on PF in BEPs and BERs

**FIGURE 5:**
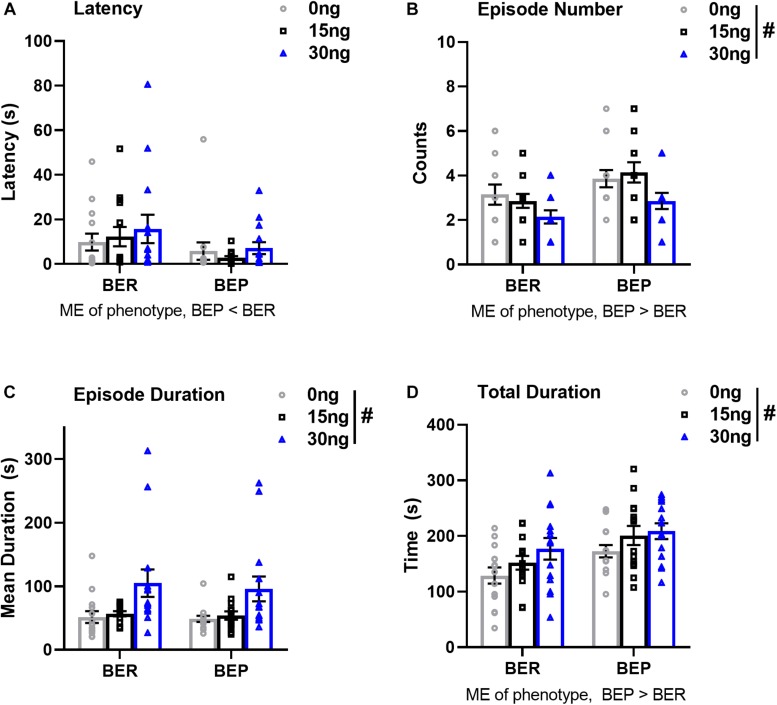
Pharmacological inactivation of the mPFC alters select structural components of PF consumption in BEP and BER rats. Values in **(A–D)** represent feeding behavior scores observed during the first 1 h of access to PF; error bars represent 1 SEM. ME, main effect; ^#^*p* < 0.05, main effect of drug.

There were no significant drug^∗^phenotype interactions for any score of feeding behavior, suggesting that the effect of mPFC inactivation on select components of feeding behavior was comparable between BEPs and BERs. First, the ANOVA for latency to begin consuming PF revealed no main effect of drug (*F*(2,52) = 0.64, *p* = 0.53), but a significant main effect of phenotype (*F*(1,26) = 4.39, *p* = 0.04, BEP<BER, Figure 5A and Table 7). The ANOVA for feeding episode number also revealed a significant main effect of phenotype (*F*(1,26) = 8.12, *p* = 0.009, BEP>BER), as well as a significant main effect of drug (*F*(2,52) = 4.62, *p* = 0.01, Figure 5B and Table 7). Specifically, inactivation of the mPFC also led to a significant decrease in the number of episodes of PF intake in both BEPs and BERs, with fewer episodes of PF intake following infusion of 30 ng muscimol as compared to saline (*p* = 0.02). The ANOVA for mean feeding episode duration also revealed a significant main effect of drug (*F*(2,52) = 9.38, *p* = 0.003, Figure 5C and Table 7), with significantly longer episodes of PF consumption following infusion of 30 ng muscimol as compared to both saline (*p* = 0.01) and 15 ng muscimol (*p* = 0.01). Mean feeding episode duration did not differ between BEPs and BERs (*F*(1,26) = 0.20, *p* = 0.66). Finally, the total amount of time spent consuming PF, over the full 1 h test, also increased as a function of drug dose (*F*(2,52) = 5.72, *p* = 0.006, Figure 5D and Table 7), with more time spent consuming PF following infusion of 30 ng muscimol as compared to saline (*p* = 0.01). The total amount of time spent consuming PF was also significantly higher in BEPs than in BERs (*F*(1,26) = 7.03, *p* = 0.01).

**TABLE 7:** Experiment 2, scores of feeding behavior following pharmacological inactivation of the mPFC in BEP and BER female rats.

	**Mean**			**Drug**	**Phenotype**
**Variable**	**(SE)**			**ME**	**ME**
	**Saline**	**15 ng**	**30 ng**	***F*(2,52)**	***F*(1,26)**
**Latency (s)**					
BER	9.83	12.30	15.77	0.64^∗∗^	4.39^∗∗^
	(5.33)	(6.12)	(9.00)		
BEP	5.79	2.83	7.11		
	(5.50)	(1.00)	(3.76)		
**Mean feeding episode duration (s)**					
BER	51.44	56.81	104.83	9.38^∗∗^	0.20
	(13.29)	(10.40)	(30.48)		
BEP	48.64	53.94	95.90		
	(6.90)	(5.71)	(27.60)		
**Mean feeding episode # (counts)**					
BER	3.14	2.86	2.14	4.62^∗∗^	8.12^∗∗^
	(0.64)	(0.44)	(0.42)		
BEP	3.86	4.14	2.86		
	(0.55)	(0.64)	(0.51)		
**Total duration of feeding (s)**					
BER	128.94	151.88	176.99	5.72^∗∗^	7.03^∗∗^
	(20.52)	(17.60)	(27.50)		
BEP	172.70	200.96	208.74		
	(15.90)	(24.21)	(20.07)		

*ME, main effect; ^∗^*p* < 0.05; ^∗∗^*p* < 0.01.*

**FIGURE 6:**
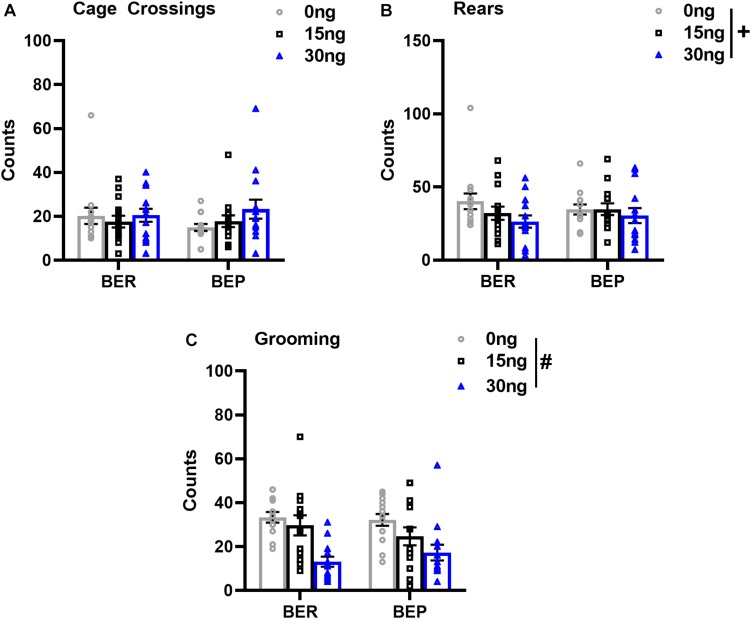
Changes in locomotor and grooming behavior in BEP and BER rats following pharmacological inactivation of the mPFC. Values in **(A–C)** represent locomotor and grooming scores observed during the first 30 min of access to PF; error bars represent 1 SEM; ^#^*p* < 0.05, ^+^*p* < 0.09, main effect of drug.

As expected, there were no significant drug^∗^phenotype interactions for any score of locomotor or grooming behavior, suggesting that the effect of mPFC inactivation on non-feeding related behaviors was also comparable between both BEPs and BERs. First, the number of cage crossings did not change as a function of drug dose (*F*(2,52) = 1.56, *p* = 0.21), and did not differ between BEPs and BERs (*F*(1,26) = 0.06, *p* = 0.79, Figure 6A), suggesting that general ambulation was unaffected by inactivation of the mPFC. On the other hand, mixed design ANOVAs did reveal a statistical trend toward a decrease in the total number of rears as a function of drug dose (*F*(2,52) = 2.98, *p* = 0.06, Figure 6B), suggesting that inactivation of the mPFC led to a general decline in exploratory behavior. There were no differences between BEPs and BERs in total rear counts (*F*(1,26) = 0.01, *p* = 0.93). Inactivation of the mPFC also led to a significant decline in the total number of grooming counts (*F*(2,50) = 15.66, *p* < 0.001, Figure 6C), with reduced grooming behavior following infusion of 30 ng muscimol as compared to both saline (*p* < 0.001) and 15 ng muscimol (*p* = 0.007); grooming counts did not differ between BEPs and BERs (*F*(1,25) = 0.05, *p* = 0.82). Thus, overall, our analysis of locomotor and grooming behavior suggests that inactivation of the mPFC caused both BEPs and BERs to spend less time engaged in non-feeding related behaviors without any undue effect on general motor function.

## Discussion

Here we provide preliminary evidence that lower, PF-induced activation of mPFC excitatory neurons may contribute to weaker, mPFC-mediated control over PF intake in BEP female rats. In Experiment 1, we show that the vast majority of PF-activated neurons within the mPFC are of the excitatory phenotype, regardless of binge phenotype, but that the magnitude of excitatory neuron responsiveness to PF is, in fact, lower in BEPs than in BERs. In Experiment 2, we demonstrate that although the mPFC functions as a behavioral “brake” over PF intake in both BEP and BER females, the strength of the mPFC-mediated behavioral brake over PF intake appears to be weaker in BEPs than in BERs. Thus, taken together, our data suggest that reduced responsiveness of mPFC excitatory neurons in the presence of PF may render the mPFC less able to adequately limit PF intake in female rats that are prone to binge eating. As such, differential engagement of mPFC excitatory neurons in the presence of PF may be an etiologic factor in the development of binge eating proneness in female rats.

In experiment 1, our analysis of Fos expression in Satb2^+^ neurons, a marker for excitatory projection neurons of the mPFC (Alcamo et al., 2008; Huang et al., 2013; Gaykema et al., 2014), revealed that most PF-activated (i.e., Fos-expressing) neurons in the mPFC are co-localized with Satb2 in both BEPs and BERs. An average of ∼85% of all Fos^+^ neurons were Satb2^+^ in both phenotypes, while the remainder of the Fos-expressing neurons were co-localized with inhibitory neuron markers. The large degree of excitatory neuron responsiveness to PF was an expected finding, given that (1) 80–90% of all neurons in the rodent mPFC are of the excitatory neuron phenotype (Giustino and Maren, 2015), and (2) most of the neurons within the mPFC that are responsive to high fat PFs, at least in male mice, are excitatory neurons as well (Gaykema et al., 2014). Thus, our data extend previous results from other labs by demonstrating that most mPFC neurons that are activated by a high-fat/high-sugar PF source in female rats are also of the excitatory neuron phenotype.

Despite a lack of BEP vs. BER differences in the *proportions* of Fos^+^ neurons that are of the excitatory neuron phenotype, the total number of Fos-expressing excitatory neurons was lower in BEPs than in BERs in the ventral half (PL and IL cortices) of the mPFC: BEPs had a substantially lower number of Fos-expressing excitatory neurons than BERs in the PL cortex and a significantly lower number of Fos-expressing excitatory neurons than BERs in the IL cortex. Thus, the reduced magnitude of excitatory neuron responsiveness to PF in the ventral mPFC of BEPs vs. BERs supports our primary hypothesis that reduced excitatory neuron responsiveness to PF is associated with binge eating proneness.

In experiment 2, pharmacological inactivation of the mPFC led to a significant increase in PF intake in both BEPs and in BERs, but the magnitude of the increase in PF intake was notably stronger in BEPs than in BERs. This was most apparent in our data set through our effect size estimates for the comparison of 1 h PF intake following an infusion of saline to 1 h PF intake following infusion of the higher dose of muscimol: the effect size for this comparison in BEPs was almost double that in BERs. Thus, despite non-significant drug^∗^phenotype interactions for PF intake following pharmacological inactivation of the mPFC, our data demonstrate that suppression of neuronal activity within the mPFC yields a substantially larger increase in PF intake in BEPs as compared to BERs. Mechanistically, the rise in PF intake following inactivation of the mPFC seen in both phenotypes is likely a consequence of dis inhibition of the nucleus accumbens: the nucleus accumbens provides strong “go” signals for hedonically driven feeding (Berridge, 2009) and glutamatergic input from excitatory projection neurons of the mPFC into the nucleus accumbens suppresses food reward (Maldonado-Irizarry et al., 1995; Kelley and Swanson, 1997; Stratford et al., 1998; Richard and Berridge, 2013). As such, if the mPFC regulates PF intake by suppressing accumbens-mediated, “go” signals in favor of PF consumption, then the larger rise in PF intake seen in BEPs following inactivation of the mPFC may reflect between-phenotype differences in the strength of baseline, glutamatergic input from the mPFC to the nucleus accumbens during PF exposure. In experiment 1, we demonstrated that mPFC, excitatory neuron responsiveness to PF is lower in BEPs than in BERs, which may reflect reduced glutamatergic input from the mPFC to the nucleus accumbens during PF exposure in BEPs. Thus, binge eating proneness may arise from a weaker, mPFC-mediated “brake” over PF intake due to reduced, mPFC glutamatergic tone within the nucleus accumbens during PF exposure.

Finally, our behavioral analysis of feeding behavior in experiment 2 revealed two notable findings. First, in both BEPs and BERs, inactivation of the mPFC induced a substantial shift in the time spent consuming PF relative to other home-cage behaviors: BEPs and BERs engaged in fewer but longer episodes of PF intake, they spent more time, overall, engaged in PF intake, and, consequentially, they spent less time engaged in other, more “routine,” home cage behaviors. That is, inactivation of the mPFC shifted behavioral strategies toward favoring PF consumption while simultaneously reducing both home-cage exploratory behaviors (i.e., rears) and overall grooming behavior (i.e., grooming counts). Previous work in male rats has demonstrated virtually identical outcomes, in terms of the overall structure of PF intake (i.e., lengthened bouts of consumption) following inactivation of the mPFC (Baldo et al., 2015). Baldo et al. (2015) noted that the lengthened bouts of PF consumption likely reflect a diminution in mPFC-mediated temporal control over PF consumption (Baldo et al., 2015), given that neuronal activity within the mPFC regulates the onset and offset of basic consummatory behaviors (Horst and Laubach, 2013). That is, the mPFC likely controls PF intake by regulating “on” and “off” signals for the initiation and cessation of bouts of PF intake. In this regard, neuronal output from the mPFC appears to regulate how long both BEPs and BERs engage in PF consumption relative to other, perhaps more advantageous, behaviors (i.e., exploring the environment, grooming).

Second, our behavioral analysis of feeding behavior also demonstrated that BEPs consistently approached the PF dish sooner and engaged in more total bouts of PF intake than did BERs. Data from studies of licking microstructure, using rats identified as being prone or resistant to binging on high-fat/high-sugar liquid solutions, suggest that shorter latencies to initiate licking bouts of a highly palatable solution, and more frequent licking bouts overall reflect greater motivation for consumption of the palatable solution (Lardeux et al., 2013; Calvez and Timofeeva, 2016; Johnson, 2017). We certainly cannot make a direct comparison between the more precise measurements of licking microstructure and the scores of feeding behavior used in our study. However, if the neurobiological features of licking microstructure extend to the ingestion of solid PFs, then our data suggests that BEPs are more motivated to consume PF than BERs; they take significantly less time to start consuming PF and they initiate more episodes of PF consumption than do BERs. Of note, the BEP vs. BER differences in scores of latency and episodes of PF intake were unaffected by inactivation of the mPFC, suggesting that the greater motivation for PF intake reflected by BEPs is likely driven by brain regions and substrates outside of the mPFC.

Despite the strengths of our study, we note important limitations that warrant comment. In experiment 1, we demonstrate greater Fos expression in the mPFC following PF exposure as compared to a non-PF, control stimulus, yet we did not specifically analyze inhibitory vs. excitatory neuronal responsiveness to the non-PF, control stimulus. We restricted our analysis of inhibitory and excitatory neuronal Fos expression to the PF stimulus in BEPs and BERs, as we have consistently shown that, behaviorally, BEPs and BERs differ only in PF intake and not in chow intake. As such, we were primarily interested in identifying specific neural correlates within the mPFC (i.e., excitatory vs. inhibitory neuronal responsiveness) that are most directly related to the known behavioral variable that differentiates a BER from a BEP. In the future, however, it will be important to verify that the BEP vs. BER difference in mPFC excitatory vs. inhibitory neuronal responsiveness is specific to PF and is not generalizable to other non-palatable food sources (i.e., standard rat chow).

Further, in experiment 2 our intra-mPFC muscimol infusions target, more generally, the ventral half of the mPFC, rather than specifically the PL vs. IL sub-regions of the mPFC. First, we chose to target a larger region of the mPFC here in order to maximize BEP vs. BER sample sizes for our study. Second, previous work in our lab has shown that neuronal responsiveness to PF is greater in BEP females vs. BER females in both the PL and the IL (i.e., the ventral mPFC), so we were primarily interested in determining how inactivation of this broader region of the mPFC, known to be associated with binge eating proneness in our lab, affected PF intake in BEPs and BERs (Sinclair et al., 2015). Going forward, it will be necessary to specifically target the PL and the IL cortices separately in each binge phenotype, given that the PL has been shown to activate, while the IL has been shown to inhibit reward seeking in the broader context of appetitive and motivated behaviors related to food intake (Balleine and Dickinson, 1998; Coutureau and Killcross, 2003; Heidbreder and Groenewegen, 2003; Tran-Tu-Yen et al., 2009; Gourley and Taylor, 2016).

In summary, our data provide preliminary evidence that lower, PF-induced activation of mPFC excitatory neurons is associated with, and may contribute to, binge eating proneness in female rats. Ultimately, our data suggests that the degree to which excitatory neurons of the mPFC can limit PF intake may differ as a function of binge eating phenotype in female rats: binge eating proneness appears to be associated with reduced, mPFC-mediated behavioral control over PF intake. Going forward, it will be important to identify the downstream, neural substrates by which the mPFC regulates PF consumption, to more fully understand the specific, circuit-level mechanisms by with the mPFC exerts executive control over PF intake and binge eating within the female sex.

## Data Availability Statement

All datasets generated for this study are included in the article/Supplementary Material.

## Ethics Statement

The animal study was reviewed and approved by Michigan State University Institutional Animal Care and Use Committee.

## Author Contributions

ES designed and performed all experiments, analyzed the experimental data, and prepared all figures and tables. KK and CS assisted with experimental design development and data interpretation, and critiqued the manuscript. All authors assisted in writing the manuscript, and read and approved the final manuscript.

## Conflict of Interest

The authors declare that the research was conducted in the absence of any commercial or financial relationships that could be construed as a potential conflict of interest.

## Footnotes

1 Overall, results from Experiment 1 were largely similar when analyses were run using only those BEPs and BERs that were identified using more stringent criteria (i.e., 4/6, 5/6, or 6/6 in top or bottom tertile of PF intake, See Supplementary Table S1). Thus, the 3/6 criteria was used, here, to maximize sample sizes.

2 As in experiment 1, results from experiment 2 were unchanged when using only those BEPs and BERs identified using more stringent criteria (i.e., 4/6, 5/6, 6/6 tertile criteria) during the initial feeding test period (See Supplementary Table S3).

3 Equation for calculating percent change in PF intake: (PF after muscimol – PF after saline)/PF after saline ^∗^100. We used each rat’s individual amount of PF intake after saline, rather than the group means, in the calculation.
