# Supplementary figures and images for: Reduced Medial Prefrontal Control of Palatable Food Consumption Is Associated With Binge Eating Proneness in Female Rats
Source: Front Behav Neurosci. 2019 Oct 31;13:252. doi: 10.3389/fnbeh.2019.00252 (PMC6834655; doi:10.3389/fnbeh.2019.00252)

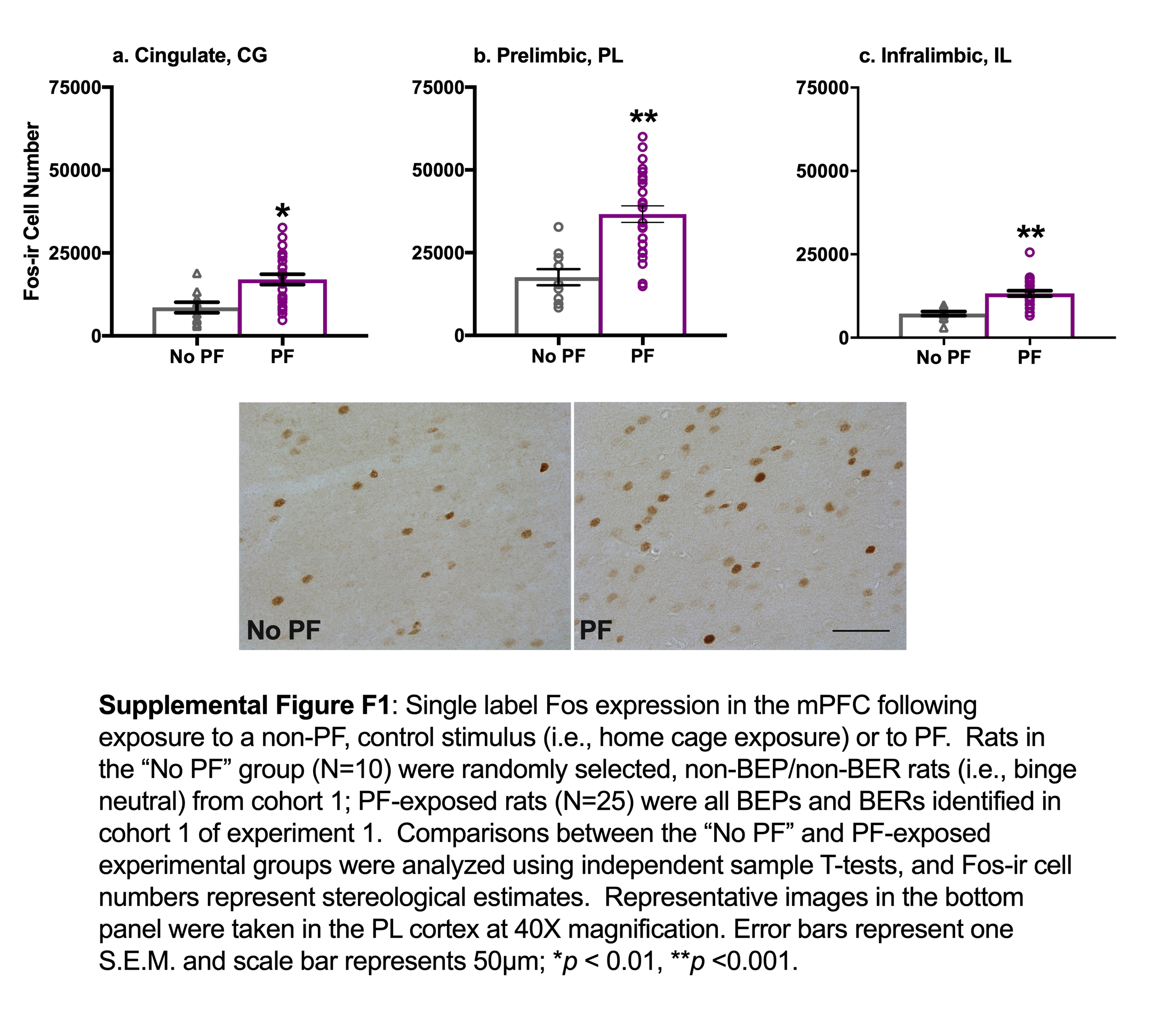

Supplement: Supplementary file 1 [file Image_1.tiff]
