# Supplementary material for: Reduced Medial Prefrontal Control of Palatable Food Consumption Is Associated With Binge Eating Proneness in Female Rats
Source: Front Behav Neurosci. 2019 Oct 31;13:252. doi: 10.3389/fnbeh.2019.00252 (PMC6834655; doi:10.3389/fnbeh.2019.00252)
Supplement: Supplementary file 2 [file Table_1.DOCX]

| **Supplemental Table S1**: BEP vs. BER differences in the total number of Fos^+^/Satb2^+^ neurons in the mPFC, using different tertile criteria for identifying BEPs and BERs | | | | |
| --- | --- | --- | --- | --- |
| Variable | Mean # (S.E.) | BEP vs. BER Mean Comparisons | | |
|  |  | *F* (df) |  | Cohen’s *d* |
| Cingulate, Fos^+^/Satb2^+^ |  |  |  |  |
| BER | 206.12 (46.65) | 0.32 (2, 22) |  | 0.25 |
| BEP | 170.39 (37.16) |  |  |  |
| Prelimbic, Fos^+^/Satb2^+^ |  |  |  |  |
| BER | 400.80 (69.37) | 0.40 (2, 22) |  | 0.28 |
| BEP | 341.20 (55.25) |  |  |  |
| Infralimbic, Fos^+^/Satb2^+^ |  |  |  |  |
| BER | 153.64 (21.48) | 0.92 (2, 19) |  | 0.45 |
| BEP | 126.85 (15.79) |  |  |  |
| Note: BEP and BER rats are those rats who fell into the top or bottom tertile of 4hr PF intake, respectively, on 4/6, 5/6, or 6/6 of the feeding tests. Means represent ANCOVA adjusted means; N = 10 BER, N = 15 BEP | | | | |
