# Supplementary material for: Reduced Medial Prefrontal Control of Palatable Food Consumption Is Associated With Binge Eating Proneness in Female Rats
Source: Front Behav Neurosci. 2019 Oct 31;13:252. doi: 10.3389/fnbeh.2019.00252 (PMC6834655; doi:10.3389/fnbeh.2019.00252)
Supplement: Supplementary file 3 [file Table_2.DOCX]

| **Supplemental table S2a**: Primary antibodies used for single and double-label immunohistochemical protocols in experiment 1 | | | | |
| --- | --- | --- | --- | --- |
| Peptide/Protein | Primary AB | Manufacturer | Dilution | Incubation |
| Fos  (single label) | Rabbit polyclonal | Millipore | 1: 10,000 | 48hr |
| Fos  (double label) | Rabbit polyclonal | Santa Cruz, Sc-52 | 1: 10,000 | 48hr |
| Satb2 | Mouse monoclonal | Abcam, SATBA4B10 | 1:200 | 48hr |
| PV | Mouse monoclonal | Sigma, PARV-19 | 1: 100,000 | 48hr |
| VIP | Rabbit polyclonal | Immunostar, #20077 | 1:4000 | 48hr |
| SOM | Rat polyclonal | Millipore, MAB354 | 1:100 | 72hr |

| **Supplemental table S2b**: Secondary antibodies and visualization for all single and double-label immunohistochemical protocols in experiment 1 | | | | |
| --- | --- | --- | --- | --- |
| Peptide/Protein | Secondary AB | Dilution | Incubation | Visualization |
| Fos  (single label) | Goat anti-rabbit IgG  (Vector labs) | 1:500 | 1hr | DAB |
| Fos  (double label, brightfield | Goat anti-rabbit IgG  (Vector labs) | 1:500 | 1hr | Nickel DAB |
| Fos (immunofluorescence) | Goat anti-rabbit IgG  Cy3-streptavidin  (Jackson Labs) | 1:1000 | 2hr | TRITC filter |
| Satb2 | Cy2 conjugated  goat anti-mouse IgG  (Jackson Labs) | 1:500 | 24hr | FITC filter |
| PV | Goat anti-mouse IgG (Vector labs) | 1:500 | 1hr | DAB |
| VIP | Goat anti-rabbit IgG (Vector Labs) | 1:500 | 1hr | DAB |
| SOM | Goat anti-rat IgG (Vector labs)* | 1:500 | 1hr | DAB |
| *Note: incubation in goat serum included 20ug/mL goat anti-rat Fab fragment for Fos-SOM double-labeling | | | | |
