# Supplementary material for: Reduced Medial Prefrontal Control of Palatable Food Consumption Is Associated With Binge Eating Proneness in Female Rats
Source: Front Behav Neurosci. 2019 Oct 31;13:252. doi: 10.3389/fnbeh.2019.00252 (PMC6834655; doi:10.3389/fnbeh.2019.00252)
Supplement: Supplementary file 4 [file Table_3.DOCX]

| **Supplemental Table S3:** PF intake following pharmacological inactivation of the mPFC in BEPs and BERs, using different tertile criteria for identifying BEPs and BERs | | | | | |
| --- | --- | --- | --- | --- | --- |
| Variable | Mean (S.E.) | | | Drug  M.E. | Phenotype  M.E. |
|  | Saline | 15ng | 30ng | *F* (2, 48) | *F* (1, 24) |
| PF intake, 1hr (g) |  |  |  |  |  |
| BER | 2.92 (0.27) | 3.12 (0.27) | 3.67 (0.41) | **9.28**** | **31.22**** |
| BEP | 4.69 (0.27) | 5.04 (0.36) | 6.32 (0.50) |  |  |
| PF intake, 4hr (g) |  |  |  |  |  |
| BER | 5.13 (0.36) | 5.34 (0.25) | 5.12 (0.38) | 1.27 | **43.65**** |
| BEP | 8.17 (0.52) | 7.73 (0.50) | 8.89 (0.41) |  |  |
| Note: BEP and BER rats are those rats who fell into the top or bottom tertile of PF intake, respectively, on 4/6, 5/6, or 6/6 of the feeding tests during the initial feeding test period; N = 13 BER, N = 13 BEP; M.E., main effect; ***p* < 0.01 | | | | | |
