# Supplementary material for: Reduced Medial Prefrontal Control of Palatable Food Consumption Is Associated With Binge Eating Proneness in Female Rats
Source: Front Behav Neurosci. 2019 Oct 31;13:252. doi: 10.3389/fnbeh.2019.00252 (PMC6834655; doi:10.3389/fnbeh.2019.00252)
Supplement: Supplementary file 5 [file Table_4.DOCX]

| **Supplemental Table S4**: Experiment 1, total numbers of Fos^+^ neurons co-localized inhibitory neuron markers PV, SOM, and VIP in the mPFC of BEPs and BERs | | |
| --- | --- | --- |
| Neuronal Marker | Mean # (S.E.) | BEP vs. BER  Mean Comparisons |
|  |  | Cohen’s *d* effect sizes |
| *Number Fos^+^/PV^+^* | |  |
| Cingulate |  |  |
| BER | 4.96 (1.94) | 1.77 |
| BEP | 1.82 (1.29) |  |
| Prelimbic |  |  |
| BER | 31.70 (6.51) | 0.98 |
| BEP | 19.65 (4.33) |  |
| Infralimbic |  |  |
| BER | 20.93 (3.93) | 0.48 |
| BEP | 18.63 (2.61) |  |
| *Number Fos^+^/SOM^+^* | |  |
| Cingulate |  |  |
| BER | 76.99 (20.97) | 0.68 |
| BEP | 114.61 (16.90) |  |
| Prelimbic |  |  |
| BER | 175.13 (38.36) | 0.42 |
| BEP | 210.81 (30.91) |  |
| Infralimbic |  |  |
| BER | 85.39 (15.22) | 0.88 |
| BEP | 118.03 (12.27) |  |
| *Number Fos^+^/VIP^+^* | |  |
| Cingulate |  |  |
| BER | 23.21 (5.67) | 0.04 |
| BEP | 22.59 (6.23) |  |
| Prelimbic |  |  |
| BER | 42.62 (7.65) | 0.44 |
| BEP | 33.67 (7.65) |  |
| Infralimbic |  |  |
| BER | 17.24 (5.90) | 0.02 |
| BEP | 16.90 (5.90) |  |
| Note: Means represent ANCOVA-adjusted means using PF consumed prior to sacrifice as the covariate. Effect sizes were calculated using ANCOVA-adjusted means. | | |
